# Zebrafish as a Model to Study Vascular Elastic Fibers and Associated Pathologies

**DOI:** 10.3390/ijms23042102

**Published:** 2022-02-14

**Authors:** Marie Hoareau, Naïma El Kholti, Romain Debret, Elise Lambert

**Affiliations:** Laboratoire de Biologie Tissulaire et Ingénierie Thérapeutique (LBTI), UMR CNRS 5305, Institut de Biologie et Chimie des Protéines, Université Lyon 1, 7, Passage du Vercors, CEDEX 07, F-69367 Lyon, France; naima.elkholti@ibcp.fr (N.E.K.); romain.debret@ibcp.fr (R.D.)

**Keywords:** zebrafish, elastic fibers, tropoelastin, elastinopathies, fibrillinopathies, cardiovascular diseases

## Abstract

Many extensible tissues such as skin, lungs, and blood vessels require elasticity to function properly. The recoil of elastic energy stored during a stretching phase is provided by elastic fibers, which are mostly composed of elastin and fibrillin-rich microfibrils. In arteries, the lack of elastic fibers leads to a weakening of the vessel wall with an increased risk to develop cardiovascular defects such as stenosis, aneurysms, and dissections. The development of new therapeutic molecules involves preliminary tests in animal models that recapitulate the disease and whose response to drugs should be as close as possible to that of humans. Due to its superior in vivo imaging possibilities and the broad tool kit for forward and reverse genetics, the zebrafish has become an important model organism to study human pathologies. Moreover, it is particularly adapted to large scale studies, making it an attractive model in particular for the first steps of investigations. In this review, we discuss the relevance of the zebrafish model for the study of elastic fiber-related vascular pathologies. We evidence zebrafish as a compelling alternative to conventional mouse models.

## 1. Introduction

Elasticity is important for the proper function of multiple organs, such as skin, lungs, blood vessels, tendons, and elastic cartilages [1]. Concerning the cardiovascular system, elasticity is mostly important in elastic arteries to smooth the pulsatile blood flow coming from the heart into a continuous flow in order to protect the organs and provide an optimal and continuous perfusion of peripheral tissues. Indeed, elastic arteries store elastic energy during systole and are able to deliver this energy back during diastole. This is known as the Windkessel effect. It is the elastic component of the vessel wall that endows it with such mechanical behavior [2].

Elasticity is mainly provided by elastic fibers in tissues. Elastic arteries are therefore fitted with a high content of elastic fibers to allow them to fulfill their role and cope with high cardiac blood pressure [2]. In contrast, muscular arteries do not need as much elasticity, as they serve rather to distribute the blood around the whole body and regulate hemodynamics by vasomotion of smooth muscle cells [3]. Elastic fibers are composed mainly of elastin (ELN), a highly hydrophobic cross-linked polymer, surrounded by fibrillin-rich microfibrils [4]. Elastin arises from the assembly of its monomeric protein precursor, tropoelastin, which is cross-linked during elastogenesis to form mature elastic fibers [4,5].

The study of vascular elastic fibers is important as several pathologies are linked to vascular deficiencies in elasticity, either from genetical or acquired causes. These pathologies are characterized by a loss of elastic fibers either regarding their quantity and/or their quality [6,7]. The term *fibrillinopathy* is used whenever the pathology affects the microfibrillar part of the elastic fibers, whereas the term *elastinopathy* is restricted to diseases directly affecting elastin. The major symptoms that arise from the lack of elasticity in vessels are due to the rigidification of the vessel wall, a condition that is also referred to as arteriosclerosis [8]. The vessel gets stiffer and weaker, leading to typical cardiovascular outcomes such as hypertension, aneurysm, dissection, or stenosis [6,7].

The zebrafish (*Danio rerio*) is a model organism widely used in biological research. It is a fresh water fish, part of the Cyprinids family in the ray-finned fish class [9]. Zebrafish and human genomes share around 70% homology. Moreover, 82% of morbidity-associated genes in human have been linked to at least one ortholog in the zebrafish [10]. These similarities support the relevance of the model, but the advantages of zebrafish are also practical. First, the zebrafish is a small organism and requires less infrastructure and nursing than mammalian models such as rodents. Their generation time is longer, approximately three to four months compared to 1.5 months for mice, but the descendants are far more numerous, with hundreds of eggs by clutch spawn every two or three days [9]. From a biological point of view, the zebrafish also offers the possibility to easily observe embryogenesis processes as embryos develop ex utero and are transparent during the first period of their life.

Zebrafish are also well adapted for genetic manipulations, with thousands of transgenic mutated strains or fluorescent reporter lines available. Numerous techniques have been developed, such as morpholino oligonucleotides, which allow the knockdown of targeted genes [11], but also genome-editing approaches based on zinc finger nucleases (ZFNs), transcription activator-like effector nucleases (TALENs) [12] and more recently, the clustered regularly interspaced short palindromic repeats (CRISPR/Cas9) system [13,14]. The CRISPR technique has also been developed to perform knockins and conditional knockouts in the zebrafish [15,16]. These modifications can be helpful to implement models with relevant characteristics of human diseases, such as specific variants associated with pathologies [17], restriction of genetic modifications to a precise time period and/or location [18], and also to avoid the compensations that are often associated with knockout experiments [19].

A chemical mutagenesis approach based on 1-ethyl-1-nitrosourea (ENU)-induced random mutations has also been smartly exploited by the Zebrafish Mutation Project [20] to generate knockout (KO) alleles of all protein-coding genes and analyze their phenotype [21]. All these techniques are efficient tools to analyze gene function in zebrafish. However, most of the zebrafish genes are present in more than one copy because of a whole genome duplication that occurred during teleost evolution history [22]. This is not a problem per se, but the investigation of potential paralogs is necessary.

Zebrafish are also highly suitable for large scale testing. For example, the model is widely used in toxicity tests [23,24,25] and in drug or therapeutic molecule discovery [26,27].

The use of animal models to study vascular fibrillinopathies and elastinopathies implies two prerequisites: the presence of comparable vascular elastic fibers in the model and the similarity of the cardiovascular system, which should react, at least to some extent, in the same way than in human when missing elastic fibers and/or elastin. To evaluate the relevance of the zebrafish model regarding this concern, we discuss in this review the basic differences between zebrafish and human tropoelastins, with respect to their protein sequences and the duplication event that conferred two tropoelastin genes to the zebrafish. We then describe the cardiovascular system of the zebrafish, with a particular emphasis on its composition in elastic fibers, even if only a few studies have approached this subject. We also present histological sections of adult zebrafish heart and vessels, showing their elastin content and organization in cardiovascular tissues. Lastly, we give an overview of already existing zebrafish models of vascular pathologies involving elastic fibers, and discuss the model relevance in this physiopathological field.

## 2. Tropoelastin in the Zebrafish

**Figure 1:**
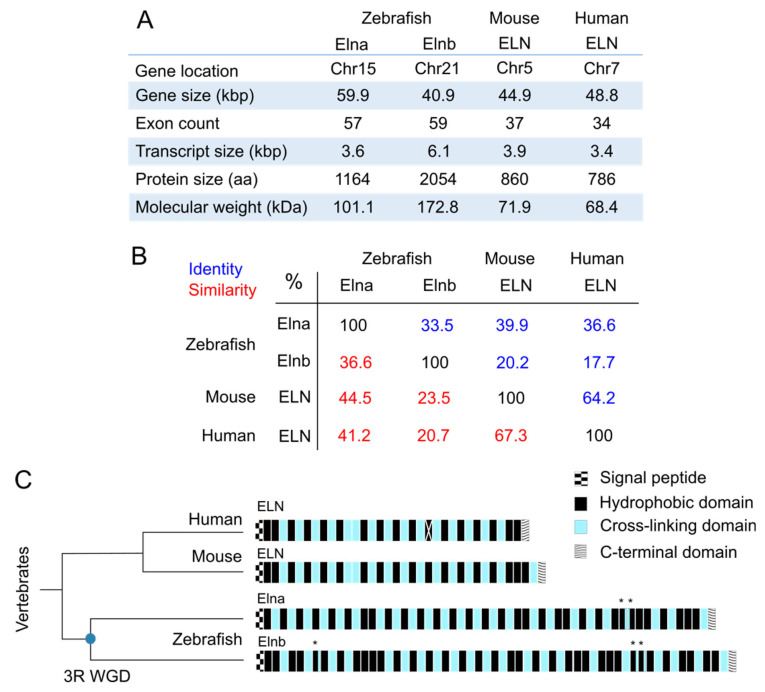
Comparison of tropoelastin sequences from zebrafish (*Danio rerio*), mouse (*Mus musculus*) and human (*Homo sapiens*). (**A**) Main characteristics of the four tropoelastins previously mentioned; kbp = kilobase pairs; kDa = kilodalton; aa = amino acid; Chr = chromosome; Data from GenBank, RefSeq NCBI and UniProt; (**B**) matrix table of the identity and similarity percentages resulting from the alignment of tropoelastin protein sequences from zebrafish (Elna TR|Q0Q5Z1|Q0Q5Z1_DANRE, Elnb TR|Q0Q5Z0|Q0Q5Z0_DANRE), mouse (ELN SP|P54320|ELN_MOUSE) and human (ELN SP|P15502|ELN_HUMAN) from UniProt, with the EMBOSS-Needle tool from EMBL-EBI; (**C**) representation of tropoelastin domains in human (ELN), mouse (ELN), and zebrafish (Elna and Elnb). Zebrafish have two tropoelastins due to a duplication event, the 3rd round whole genome duplication (3R WGD), which is symbolized by a blue round on the schematic phylogenetic tree chart. Each rectangle represents a domain, the white cross on human ELN exon 22 represents the fact that it is spliced in all transcripts; * indicates domains with both hydrophobic and cross-linking characteristics; exons 13 and 14 in human ELN are not always attributed this way [32,33,34,35].

Tropoelastin is found in all vertebrates except for Agnathans, the superclass of jawless fish [28], and does not seem to exist in invertebrates as all tested specimens were devoid of it [29]. Due to a whole genome duplication (WGD), also called teleost-specific “TS” or 3rd round “3R” WGD that occurred approximately 350 million years ago and gave rise to the teleost branch, most of human genes have more than one ortholog in the zebrafish [30,31]. Therefore, zebrafish and teleost in general, have two different elastin genes named *elna* and *elnb* (or sometimes *eln1* and *eln2*) coding for two distinct tropoelastin proteins: Elna and Elnb, respectively (Figure 1).

### 2.1. Sequences

General characteristics of both tropoelastin genes and proteins in zebrafish are summarized and compared with their mouse and human orthologs in Figure 1A. The *elna* and *elnb* zebrafish paralogs are located on different chromosomes (chr), chr 15 and chr 21, respectively, but they share conserved synteny among them and with their human and mouse orthologs as demonstrated by the proximity with *LIM domain kinase 1* (*limk1*) genes in all species [36].

Protein sizes differ a lot between mammals and zebrafish. Elna and Elnb are respectively 101 kilodaltons (kDa) with 57 exons and 173kDa with 59 exons, compared with human, whose tropoelastin is around 68 kDa and composed of 34 exons. There was no obvious sign of alternative splicing reported for these zebrafish tropoelastins, so we can consider that they are indeed much bigger than their mammal counterparts. Alignments of protein sequences with EMBOSS-Needle (EMBL-EBI) revealed that Elna was closer to human tropoelastin (ELN) than Elnb. They share approximately 37% identity and 41% similarity, whereas for Elnb, around 18% identity and 21% similarity were calculated. The difference between Elna and Elnb is also very important, being even less close between each other than Elna and tropoelastins from both human and mouse (Figure 1B).

Zebrafish tropoelastin amino acid sequences share some particularities in their organization with other tropoelastin sequences from vertebrates. They are all composed of alternating hydrophobic domains and cross-linking domains (Figure 1C) [1,34]. This structure is probably important for the capacity of the protein to self-assemble and also for its elastomeric mechanical properties [4]. The C-terminal domain of the protein is highly conserved among vertebrate species. It contains a tetrabasic motif that intervenes in tropoelastin interactions with other proteins such as integrins, and is therefore important for cell adhesion [37]. There are also two cysteines that form a disulfide bond, which was shown to facilitate elastic fiber assembly [38]. These characteristics found in all described tropoelastins are likely to be the “fundamental” requirements to obtain a functional protein. However, although the presence and arrangement of hydrophobic domains are globally well conserved, the nature of these domains are very different from one species to another. It surely means that the global characteristics of domains are far more important than their precise sequences. Most of the domains in tropoelastins are encoded by specific exons. However, in zebrafish tropoelastins and particularly in Elnb, some otherwise hydrophobic domains contain cross-linking-specific motifs, resulting in “hybrid” domains, indicated by asterisks on Figure 1C. In addition, hydrophobic domains of Elnb are larger on average than those of other tropoelastins, but both zebrafish tropoelastins are less hydrophobic than mammal ones (as per the Kyte-Doolitle scale that measures hydropathy [39]), most likely due to an increase in the glycine content compared to valine and alanine [40]. Concerning the cross-linking motifs, some divergence between on one side Mammals and Aves compared to on the other side Amphibians and Fish can be noted. Whereas the most common motifs are KAAAK or KAAK in Mammals and Aves, KAAAK is not found in Amphibians and Fish and KAAK is only rare. They rather take the form of KxxK motifs with at least one of the x being a proline, a sequence rather rare in other tropoelastins (3 occurrences in human, 4 in mouse vs. 10 occurrences in Elna and 13 in Elnb, as reported by Chung et al. [34]). These KP-type motifs could also lead to different cross-links as they do not promote the formation of usual desmosine and isodesmosine cross-links [41], which were as a matter of fact reported to be less present in teleost and amphibian elastins [42].

### 2.2. Spatio-Temporal Expression

The expression of both *elna* and *elnb* mRNA peaks at six to seven days post-fertilization (dpf) and decreases at eight dpf to reach a basal level of expression at 23 dpf, which corresponds to the end of the metamorphosis stage and almost the start of the juvenile stage in zebrafish life cycle [43]. This kinetic of expression is comparable to mammals, in which the elastin gene is particularly active during the last third of fetal development and during the perinatal period. After this peak, mRNA levels decrease to reach a low constitutive level at the end of growth period [44,45].

When two similar genes coexist, one of them can progressively disappear or mutate enough to give a protein with new functions. If the mutation is advantageous, it is more likely to persist and become the dominant version with time. The duplicated gene has three main possible fates: (i) pseudogenization or non-functionalization: one of the copies is silenced; (ii) subfunctionalization: both copies take over some roles that were already given to the ancient protein, or sometimes the pattern of expression is distributed within both orthologs so that each one specializes in different regions; (iii) neofunctionalization: one of the copies acquires a new function [30,46]. In the case of zebrafish tropoelastins, both proteins are expressed and were detected, as expected, in elastic tissues: early in skeletal cartilage structures of the head, in the outer and inner wall of the swim bladder and in the cardiovascular system with some disparities depending on the elastin gene [43]. *elnb* was predominantly expressed in the bulbus arteriosus (BA), even if also present in vessels and in the swim bladder [43]. Specificities regarding its function still remain to be determined. However, a potential new role for Elnb has been described in favor of neofunctionalization, and will be detailed in Section 3.1.5.

To further understand Elna and Elnb deposition in cardiovascular elastic tissues, we hereafter detail the anatomy and function of zebrafish cardiovascular system.

## 3. Zebrafish Cardiovascular System and Its Elastic Components

**Figure 2:**
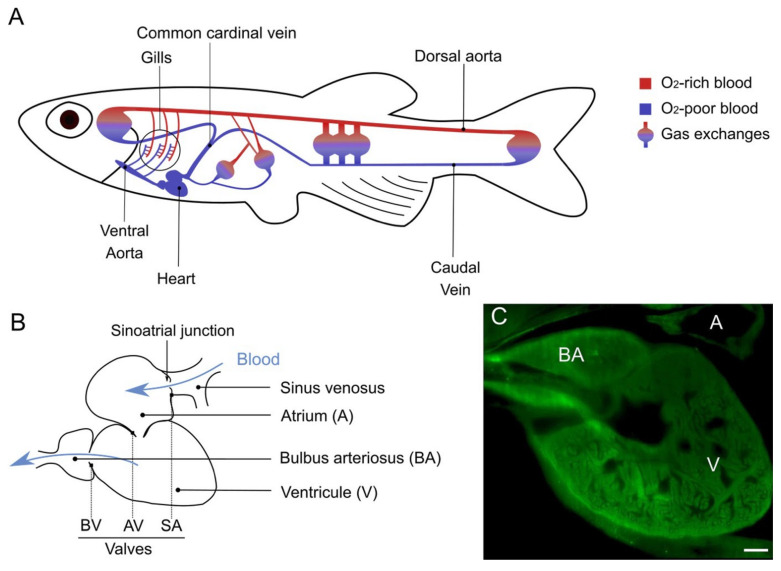
Zebrafish cardiovascular system. (**A**) Simplified illustration of the cardiovascular system of an adult zebrafish. The heart pumps deoxygenated blood to the ventral aorta, which distributes it to afferent branchial arteries, leading to gills (encircled). Once oxygenated, the blood is drained into efferent branchial arteries, to then be collected in the dorsal artery, which supplies the anterior and posterior parts of the fish. Exchanges are made in capillary beds and deoxygenated blood is returned to the heart for a new cycle. (**B**) Illustration of a zebrafish heart with its four cavities, the direction of blood is indicated by blue arrows; Valves are pointed with dotted lines; BV = bulboventricular; AV = atrioventricular; SA = Sinoatrial. (**C**) Image of an adult zebrafish heart obtained by light sheet microscopy (autofluorescence); the trabecular structure of the ventricle can be seen; Scale bar = 100 µm.

Vertebrates, from jawless fish to human, all share a basic organization of their cardiovascular systems, inherited from a common ancestor [47,48]. A simplified illustration of the zebrafish circulatory system is given in Figure 2A. Major constituents will now be described.

### 3.1. The Heart

In contrast to humans and their double circulation, zebrafish and fish in general have a simple circulation. In a double system, the systemic circulation is in parallel with the pulmonary circulation, and both oxygenated and unoxygenated blood goes through the heart, each in its dedicated side (left atrium and ventricle for the systemic circulation and right atrium and ventricle for the pulmonary circulation). In fish simple circulation, the unoxygenated blood is pumped by the heart and is led to gills, which are in series with the heart (Figure 2A) [49,50]. A fish heart is composed of two pumping chambers instead of four as in mammals: a single atrium that receives blood and a single ventricle that expulses it out. Nevertheless, the zebrafish heart is sometimes described as four-chambered. Indeed, we can distinguish four structures forming the heart: besides the atrium and the ventricle are the sinus venosus, which collects the blood from veins before pouring it in the atrium, and the bulbus arteriosus, right at the exit of the ventricle, that leads the blood from the heart to the ventral aorta (Figure 2B). These four parts of the zebrafish heart will be described in details in the next subsections, but the distinction should be made that only the atrium and the ventricle are contractile and with closing valves at both entry and exit, as chambers found in mammals are, so the other two should not be considered as “chambers” per se [47,49,50].

The heart of adult zebrafish is approximately 1.5 mm (height) × 1 mm (width), whereas an adult human heart is around 12 cm (length) × 8.5 cm (width) × 6 cm (thickness) [51,52]. Both organs are made of three layers: the endocardium, composed of endothelial cells that line heart cavities; the myocardium with all muscle cells or cardiomyocytes; and the epicardium, which is the surface of the heart, made mostly of epithelial cells and connective tissue [49]. As in other vertebrates, an acellular layer of extracellular matrix known as the cardiac jelly is present between the endocardium and the myocardium in zebrafish embryonic heart [53,54]. It is involved in heart development, playing a role in heart septation [55] and valve formation [56], not only by structural means, but also by influencing signaling pathways [57].

The blood pressure of zebrafish peaks at ~2.51 mmHg in the ventricle during systole, whereas it reaches around 120 mmHg in human. The minimum and maximum mean blood pressures in human aorta are 80 mmHg and 120 mmHg, respectively. In the zebrafish ventral aorta, the blood pressure varies from 0.8 mmHg to a little more than 2 mmHg [58]. Even if the values are far from one another, a pressure gradient along the arterial tree still exists: the highest point is in the ventricle and an important drop of pressure occurs across gills [50].

Cardiac electrophysiology is also studied a lot in zebrafish as it resembles human cardiac electrophysiology. Electrocardiogram patterns are comparable between the two species [58] and the zebrafish model shares some parameters that are closer to the human model than the mouse model. For example, their heart beats are closer (fish: 120–180 beats per minute (bpm); human: 60–100 bpm; mouse: 300–600 bpm), their cardiac phases timing is also more resembling and the nature of ion channels involved in the process is different in mice, whereas the zebrafish shares more similarities, being therefore more relevant in this regard [59].

#### 3.1.1. The Sinus Venosus

The sinus venosus (Figure 2B) collects all the unoxygenated blood that comes from the periphery by the common cardinal vein and the hepatic portal vein to send it to the atrium [58]. The sinus venosus has the thinnest wall of heart cavities [49], with almost no myocardium in the zebrafish, unlike in other fish [47]. The sinus venosus is a persistent structure in fish heart but it has a transitory equivalent in the human embryonic heart that merges with the right atrium to give the sinus venarum, the part of the atrium between the inferior and the superior vena cava [60].

The sinus venosus is also important because it contains pacemaker cells and some conductive tissue, responsible for the rhythmic contractions of the heart. More precisely, these cells were found at the sinoatrial junction (Figure 2B), corresponding to the sinoatrial node in mammals. The specialized fibers (His bundle and Purkinje fibers) that are responsible for transmitting the action potentials throughout mammalian heart tissues do not seem to be present in the zebrafish [61,62]. It was suggested that the ventricular trabeculae, visible in Figure 2C, are responsible for the conduction of the impulse instead [59,63].

#### 3.1.2. The Atrium

The atrium is mostly composed of cardiac muscle, with some extracellular collagen and elastin as well as interstitial cells. Unlike mammals, zebrafish cardiomyocytes form trabeculae, which are sorts of bundles. Mammalian myocytes are roughly rectangular whereas fish myocytes are smaller and more spindle-shaped [50,64].

#### 3.1.3. The Ventricle

**Figure 3:**
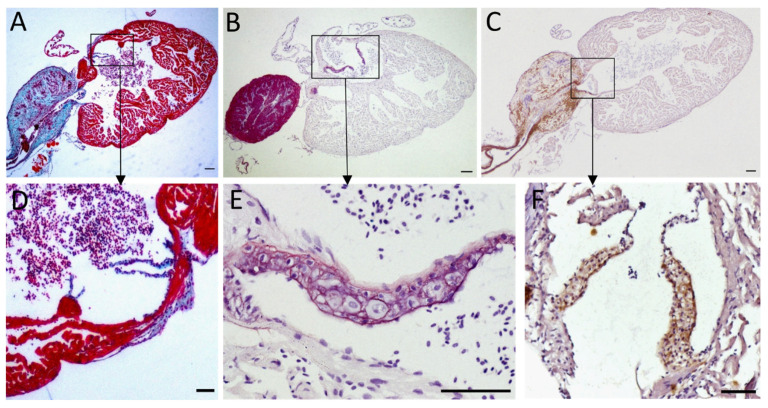
Elastin content of the zebrafish heart. Histological sections of adult zebrafish entire hearts (**A**–**C**) and cardiac valves (**D**–**F**). Sections were stained following different protocols: Masson-Goldner trichrome staining (**A**,**D**) makes muscle fibers appear in red, collagen in blue, and erythrocytes in pinkish violet; orcein (**B**,**E**) stains elastin in pink and nuclei appear in violet due to hematoxylin staining; immunohistochemistry against elastin (**C**,**F**) with elastin signal appearing in brown; (**D**,**E**) are atrioventricular valves, (**F**) shows the bulboventricular valve. Scale bars: 100 µm (**A**–**C**); 50 µm (**D**–**F**). Magnification: 40× (**A**–**C**); 200× (**D**,**F**); 00× (**E**).

The ventricle is the principal contributor to the generation of blood pressure, the atrium being less involved. The ventricle has a pyramidal shape [58] and has two distinct layers, a trabecular one of higher density than in the atrium and a compact one (Figure 2C) [49]. It is, as the atrium, mainly composed of cardiac muscle (Figure 3A–C) with a thicker wall than the atrium. The compact layer is vascularized by coronary arteries and capillaries, coming from the efferent branchial arteries [49,58]. Some coronary vessels have also been found in the trabecular layer more recently [65]. As mammalian myocytes, zebrafish myocytes are connected by gap junctions with desmosomes, allowing the synchronization of all cells to cardiac rhythm [58]. There is however no t-tubule system in the zebrafish as those found in mammals. Also, contrarily to other vertebrates that rather adjust their heart rate to modulate their blood pressure, fish can change their stroke volume to obtain the same result [50].

#### 3.1.4. Cardiac Valves

In zebrafish heart, valves are present between the sinus venosus and atrium (sinoatrial valve) [60], between the atrium and the ventricle (atrioventricular valve) and between the ventricle and the bulbus arteriosus (bulboventricular valve) (Figure 2B) [66]. They prevent the blood flow from going backwards. There are differences in the morphology and organization of valves between mammals and fish, such as (i) the absence of papillary muscle and the connection of chordae tendinae to the atrioventricular valve of the zebrafish heart; (ii) the bulboventricular valve of zebrafish, equivalent to mammal aortic valve, has only two leaflets instead of three; and (iii) as for many organs in zebrafish, its valves are capable of regeneration through the activation of transforming growth factor β (TGF-β) signaling pathway [59,66,67,68]. Nevertheless, the zebrafish has been proven useful to understand valve development and physiopathology with the help of mutants that display valvular defects [59,69]. Orcein staining and elastin immunohistochemistry enable to highlight the presence of elastin in atrioventricular and bulboventricular valves of the zebrafish heart (Figure 3D–F).

#### 3.1.5. The Bulbus Arteriosus

The bulbus arteriosus (BA) is a specialized outflow tract found in teleost fish derived from the conus arteriosus present in more “primitive” fish. It is located right at the exit of the heart, after the ventricle, forming a supplementary “pseudo-chamber” that is not found in humans or mammals. It is a pear-shaped organ, and although being inside the pericardium so that it is considered to be part of the heart, it has mostly vascular characteristics regarding histology and function [58]. Indeed, zebrafish BA contains smooth muscle cells, as do vessels, and no cardiac muscle [70]. This is a difference compared to the conus arteriosus where cardiomyocytes are found [47]. In addition, it is organized in the three characteristic layers of vessels, namely the intima, the media, and the adventitia. Unfortunately, the precise arrangement of elastic fibers and other extracellular matrix proteins in zebrafish BA is poorly described in the literature [58]. It was however described in the Yellowfin tuna (*Thunnus albacares*), which is also a teleost fish [71]. It is important to note that tunas are “high performance fish”, as they need to migrate, whereas zebrafish do not. Thus, BA description in tuna might not be transposable to zebrafish since they do not share the same way of life. In tuna BA, the intima is classically composed of a unique layer of endothelial cells. The media is mainly composed of smooth muscle cells and elastin. It is the thickest layer of the BA, representing around 90 to 95% of the organ. Real differences were noted between the organization of the vascular wall in the bulbus arteriosus compared to the ventral aorta. The ventral aorta has a traditional organization with elastin lamellae forming concentric layers, alternating with smooth muscle cells parallel to the others, as in mammal arteries. Conversely, no lamella was observed in the BA, but rather elastin “fibrils” that do not correspond to mature elastic fibers, neither in their composition, nor in their mechanical properties. A lower hydrophobic index and a lower elastic modulus of these fibrils were reported compared to fibers in the ventral aorta [71].

As explained previously, elastic arteries in mammals smooth the blood flow. They distend as they receive blood from the heart during systole, and due to their elastic properties, they can give back the energy of the deformation to compensate the drop of pressure during diastole. In the end, the pulsatile flow becomes continuous in the periphery. In the zebrafish, this role is played by the bulbus arteriosus, which acts as a Windkessel organ. Its major utility is to protect downstream organs, in particular gills that are very close to the heart (Figure 2A) [47,59,70]. Similar to elastic arteries, the bulbus arteriosus contains high amounts of elastin (Figure 3B,C) and is therefore able to act as an elastic energy reservoir [58,59]. Collagen fibrils also participate in the organization and properties of the tissue (Figure 3A). In addition, the BA can stock all of the stroke volume [72] and it was described in some fish as 30 times more compliant than aorta in mammals [71]. This high capacity of deformation is linked to a high elastin/collagen ratio, which can be about 10 times higher in the rainbow trout (*Oncorhynchus mykiss*) BA than in mammals’ aorta [71]. It goes without saying that these observations, made on other teleost fish, need to be confirmed in the particular case of the zebrafish.

As it was discussed in the first section, teleost fish have two genes encoding for two distinct tropoelastin proteins: Elastin a, the closest to human ELN and Elastin b, less resembling. It has been shown that the most expressed form in the BA was the Elnb paralog. This is coherent with the hypothesis of neofunctionalization proposed by Moriyama et al. [36], suggesting that Elnb could have evolved to fulfill a new role, being also responsible for the emergence of the bulbus arteriosus in teleost fish. Indeed, Elnb is necessary for BA cells to differentiate from cardiac precursor cells to smooth muscle cells during development. Its down-regulation leads to the apparition of ectopic cardiomyocytes in the BA and its complete absence can cause severe hypoplasia of the BA. By contrast, Elna down-regulation or absence has no real impact on the bulbus arteriosus. The particularity of the sequence of Elnb could also explain the unconventional organization of elastin in the BA and its unique mechanical properties [71]. Elnb would therefore have novel structural and signaling capacities compared to Elna, which gave rise to the teleost-specific Windkessel organ that is the bulbus arteriosus [36].

Quite interestingly, we were able to recognize zebrafish elastin in atrioventricular and bulboventricular valves and in the BA (Figure 3C,F) using an anti-elastin antibody directed against the N-terminal portion of mouse tropoelastin (#ab21600 from Abcam, Cambridge, UK). This result first highlights a broad recognition of tropoelastin among various species but we could also note that elastin signal in the BA was lower by immunohistochemistry detection than with orcein staining, a coloration enabling the visualization of elastic fibers (Figure 3B,C). This might be explained either by a lower efficiency of the antibodies for zebrafish elastin or by the recognition of only one zebrafish elastin paralog.

### 3.2. Arteries and Vessel Wall Organization

In adult zebrafish, the major arteries are the ventral aorta and the dorsal aorta. The ventral aorta has the particularity to be a dead end in zebrafish (Figure 2A), as its ramifications diverge from the side of the vessel. There are four pairs of afferent branchial arteries that lead the blood to the gill filaments, where it is oxygenated and then sent to efferent branchial arteries, which merge to give the dorsal aorta [58]. The dorsal aorta of adult zebrafish has been described in details by Miano et al. [73], giving a solid basis to understand the organization of the vascular wall in zebrafish arteries in general.

As mentioned previously, the vascular wall of zebrafish follows the same organization as vessels in other vertebrates [50]. They are usually structured in three layers: the tunica intima, media and adventitia or externa, from lumen to periphery. These layers are more evident in large vessels and are not found in capillaries and venules. The global behavior of the vessel depends on the organization of the vascular wall and its exact composition varies sensibly between vessels and can change with time.

The intima is composed of endothelial cells and a layer of extracellular matrix that forms a basement membrane. Endothelial cells are attached together by tight junctions. They serve as a barrier between blood and tissues. Some electron-dense bodies have been reported in zebrafish endothelial cells one month post-fertilization (mpf), but their function remains unknown. These organelles could also have been observed in the BA but identified as lipids at the time [58,73]. They could correspond to mammalian Weibel-Palade bodies, which contain factors important for coagulation, even if they do not present the same structure. A tunica elastica or internal elastic lamina can be seen between the intima and the media, composed mainly of elastic fibers and collagen [50,73].

**Figure 4:**
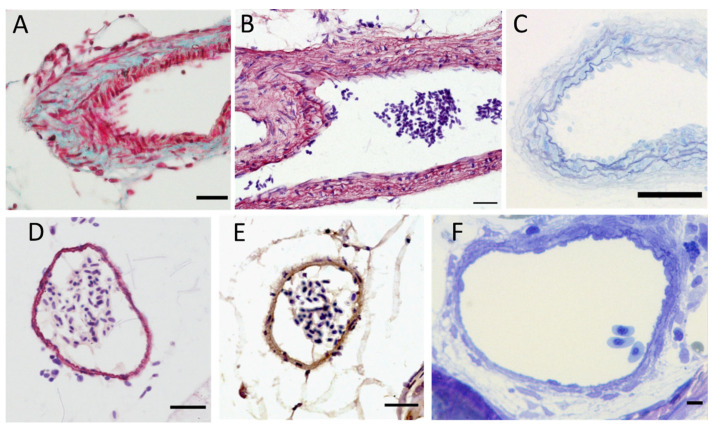
Elastin content of zebrafish major arteries. (**A**–**C**) Ventral aorta sections stained with Masson-Goldner trichrome (**A**), orcein (**B**) or by toluidine blue on a semi-thin section (**C**); scale bar = 10 µm; (**D**–**F**) dorsal aorta sections stained with orcein (**D**), by immunohistochemistry using anti-elastin antibodies (**E**) or by toluidine blue on a semi-thin section (**F**); scale bar = 10 µm (**A**,**B**); 5 µm (**C**–**F**). Magnification: 400× (**A**–**E**); 1000× (**F**).

The media contains vascular smooth muscle cells. The content in elastin/elastic fibers decreases the further we move from the heart as observed in mammals, with the highest content being in the BA (Figure 3B,C and Figure 4B–E). Generally, elastic fibers in arteries are oriented in concentric lamellae to distribute the stressing forces uniformly across the vessel wall. These lamellae alternate with a layer of smooth muscle cells, forming a functional unit that can handle pressure as long as it is in a physiological range or under [74]. In mammals, the thickness of the vessel wall varies according to blood pressure. In humans, the number of lamellae can be very high, with around 80 lamellae in the ascending aorta [75]. The ventral aorta and the bulbus arteriosus are right between the ventricle and gills, so they have to deal with the high-pressured blood flow coming from the former to protect the latter. They consequently have globally the same role as elastic arteries in mammals, even if the pressures at stake are far less important. We can count 8 to 10 lamellae at the exit of the BA (Figure 4B) and 4 to 5 lamellae further in the ventral aorta (Figure 4C). In the dorsal aorta, only one to two lamellae are visible (Figure 4D–F), which is coherent with the one or two layers of smooth muscle cells reported by Miano et al. [73].

Lastly, the adventitia is mostly made of fibroblasts and collagen, as in mammals. Collagen is important in large arteries, as it prevents the vessel from breaking under high pressure [76].

Some particularities of zebrafish arteries should also be mentioned, such as arterial valves that were found in the dorsal aorta, most likely helping to maintain unidirectional flow under low systolic pressure. In addition, the presence of melanocytes in the surrounding of vessels was observed [73].

## 4. Vascular Elastic Fiber Pathologies and Associated Zebrafish Models

The zebrafish model has already been widely used in cardiovascular research, with substantial progress made in cardiovascular development [77,78,79], angiogenesis [80], and cardiac regeneration [81,82,83]. It is also increasingly used to model human diseases in general [84,85,86] and some reviews already presented zebrafish interests for the study of cardiovascular pathologies [87,88,89,90].

Elastic fibers are composed of an elastin core interwoven in a scaffold of fibrillin-rich microfibrils. Other proteins help to assemble the fibers and participate in its structure and biological roles, such as the microfibril-associated glycoproteins (MAGP), the latent TGF-β-binding proteins (LTBP), the A disintegrin and metalloproteinase with thrombospondin motifs (ADAMTS), fibulins and elastin microfibril interface-located proteins (EMILINs) [91]. Most of the proteins involved in the formation and maintenance of elastic fibers are conserved throughout vertebrates [74]. Genetic alterations of these proteins can lead to defective fibers, most of the time making the vessel wall weaker and more prone to develop cardiovascular complications. Interestingly, some of these elastic fiber-associated pathologies are studied in the zebrafish and will be presented in the next paragraphs.

### 4.1. Elastin

Elastin is the major component of elastic fibers, accounting for about 90% of the mature fiber content. Some diseases are directly linked to a mutation in the *ELN* gene in human, such as Williams Beuren syndrome (WBS, also called Williams syndrome), non-syndromic supravalvular aortic stenosis (SVAS), or the autosomal dominant cutis laxa (ADCL) [6]. Associated cardiovascular symptoms can be more or less severe depending on the considered genetic defect [6,92].

As mentioned earlier in this review, knockdown (KD) experiments performed on zebrafish have highlighted the role of Elnb in the BA formation, but the exact role of both paralogs on vascular system has not been precisely deciphered yet. Using a combination of ENU-mutagenesis, whole exome enrichment and Illumina sequencing, in addition to a targeted CRISPR/Cas9 approach, the Zebrafish Mutation Project (ZMP) has generated a mutant archive of over 40,000 alleles, covering 60% of zebrafish protein-coding genes. In this archive, seven mutants for *elna* have been identified, among which four are currently available and two mutants for *elnb* are commercially available. However, none of these mutants have been described yet and the effects on zebrafish cardiovascular system remain to be investigated.

### 4.2. Fibrillins

The type-1 Marfan syndrome (MFS) is an autosomal dominant fibrillinopathy linked to a mutation in the *fibrillin-1* (*FBN1*) gene. It results in poorly functional elastic fibers and a disturbed regulation of TGF-β signaling pathway, as microfibrils are involved in the bio-availability of mature TGF-β. A misregulation of TGF-β signaling can lead to severe pathologies in vessels, acting on collagen and elastin metabolism as well as on the proliferation of smooth muscle cells [93,94]. MFS is a systemic condition, so that patients develop a wide variety of symptoms such as skeletal and ocular defects and, in the case of vascular defects, MFS patients exhibit a progressive dilation of the aorta with higher risk of aneurysms and dissections. Valvular leaks are also common [95].

There are four fibrillins in the zebrafish instead of the three found in humans [96]. Fibrillin-1 sequence is highly conserved in vertebrates, including the zebrafish [97]. Down-regulation of fibrillin-1 in the zebrafish using morpholino antisense oligomers was found to affect the zebrafish cardiovascular system through dilation of the caudal vein and of vessels in the head and eyes of zebrafish embryos [98]. Additionally, the mutation of the fibrillin-1 ortholog in the zebrafish by CRISPR/Cas9 technique was described recently by Yin et al. [97], with the report of interesting cardiovascular outcomes. Heterozygous *fbn1* zebrafish mutants were found to have an abnormal blood flow between the atrium and the ventricle, being also slower and less abundant compared to wild-type zebrafish. These first observations, conducted until 40 dpf, support the potential of this model for MFS research.

### 4.3. MAGPs

MAGP are also well conserved in mammalian and teleost fish. They are not structural proteins but are rather important for TGF-β signaling and are involved in a lesser extent in the Notch pathway [99]. In humans, MAGP mutations have also been associated with pathologies. MAGP-2 (*MFAP5*) haploinsufficiency has been linked to thoracic aortic aneurysms and dissections, most likely due to a dysregulation in TGF-β signaling [100]. MAGP-1 is mostly associated with obesity and metabolic syndrome, as is MAGP-2, but was not directly linked to vascular issues [99]. Different results were obtained in mice, where only double MAGP-1 and 2 homozygous mutants were showing vascular defects (aortic dilation) after four to six months of life [99]. In the zebrafish, the silencing of *mfap2* (coding for Magp-1) during embryogenesis, with a morpholino-based approach, led to vascular defects with an abnormal organization of the vessel wall, containing less elastin and some detaching cells [98]. Aortic dilations were also observed, as in the *fbn1* morphant mentioned previously. Some vascular defects were also reported by Alvarez et al. [101] in the context of retinal vasculature, with ineffective patterning of vessels, also based on an *mfap2* (Magp-1) morpholino knockdown.

### 4.4. LTBPs

LTBPs are also important contributors to TGF-β bioavailability. Moreover, LTBP-2 and 4 have been shown to play a part in the regulation of elastic fiber assembly [102]. The mutation of LTBP-2 has been linked to a form of Weill Marchesani syndrome (WMS), associated with cardiovascular defects in some patients [103]. However, most of the time, symptoms are restricted to the eye region, suggesting a compensation by the unaffected LTBPs in the other tissues [104]. Mutations in the *LTBP-4* gene have been associated with autosomal recessive cutis laxa type IC, in which cardiovascular symptoms are not predominant and reportedly restricted to peripheral pulmonary artery stenosis [105,106]. LTBP-3 and LTBP-4 losses were studied in mice models and caused cardiovascular defaults such as aortic dilations and dissections for the former and cardiomyopathies for the latter. In zebrafish, *ltbp-1* and *3* mutants have been described. The lack of Ltbp3 affected the development of the heart in zebrafish embryos, surprisingly by a decrease in TGF-β signaling [107]. A *ltbp-1* mutant was recently described and could result in a “cutis laxa-like pathology”, but no cardiovascular observations were reported so far, with mostly skin and bone symptoms [108].

### 4.5. GLUT10

Arterial tortuosity syndrome (ATS) has also been linked to an abnormal TGF-β activity that results in elongated and tortuous arteries. The formation of elastic fibers in patients suffering from ATS is impaired [109]. The disease was linked to the glucose transporter GLUT10 (*SLC2A10*), but the mechanism is not perfectly understood yet. The evolutionary conservation of this glucose transporter was confirmed by gene and protein sequence analysis, and vascular abnormalities were observed in *slc2a10* knockdown embryos, such as a reduced heart beat and incorrect vascular patterning, thus mimicking the human disease [110].

### 4.6. ADAMTSs

ADAMTS are proteins associated to microfibrils and involved in extracellular matrix remodeling. They are evolutionary conserved throughout vertebrates and even have orthologs in various invertebrates [111]. There are 19 ADAMTS proteins in mammals with different functions and activities. Some of them have been linked to cardiovascular disorders in mice models such as ADAMTS5 and 9. In human, ADAMTS5 was found to be downregulated in aneurysmal cerebral arteries [112]. Interestingly, Adamts5 and 15a were found to be strongly expressed in the zebrafish heart, but not Adamts9 [111]. Moreover, Adamts5 specific inhibition in zebrafish embryos confirmed its role in aneurysm development, as treated embryos had higher risks of developing intracranial hemorrhage than embryos from the control group [112].

### 4.7. Fibulins

Fibulins are other important actors of elastic fiber assembly. Eight isoforms are found in humans. They play a role in the structural integrity of elastic tissues, in their remodeling, and could also have regulating functions over TGF-β signaling [113]. Fibulin-1, 2, 4, and 5 are the most involved in the vasculature with a particular role in elastogenesis for the last three [114]. Mutations in fibulin-4 were linked to autosomal recessive cutis laxa type IB, associated with aortic dissection, and mutations in fibulin-5 with autosomal recessive cutis laxa type IA, associated with supravalvular aortic stenosis (SVAS) and peripheral pulmonary artery stenosis [114]. Down-regulation and overexpression of *fibulin-5* were tested in the zebrafish in the context of Charcot-Marie-Tooth neurological disease, but no effect on cardiovascular system was reported [115]. Fibulin-7 could play a role in heart formation as it was associated with a congenital heart disease due to a newly identified chromosomic deletion. A KD of *fibulin-7* in the zebrafish was tested and led to similar cardiac and craniofacial defects than those found in the human syndrome [116]. Fibulin-1 was also reported to be expressed during cardiac development near the valve regions in the zebrafish, but its exact role is not described yet [117].

### 4.8. EMILINs

In human, EMILINs are a family composed of four isoforms, which are known to play an important role in maintaining vascular homeostasis and regulating elastogenesis [118]. In the zebrafish, eight EMILINs paralogs are found, as each human member has two paralogs with conserved structures [119]. In mice, EMILIN-1 deficiency has been linked to aortic valve disease and aortic aneurysm [120]. A recent study reported that Emilin1a zebrafish morphants displayed altered locomotion and abnormal motor neurons, but no alteration in cardiovascular system was described [121]. Other Emilin mutants have been generated through the ZMP, but no study using those mutants has been reported yet.

### 4.9. Ion Metabolism

We will now discuss diseases affecting elastic fibers by a modification in ion metabolism. In general, ion metabolism is well conserved between vertebrate species and the zebrafish model has been used extensively in this field, allowing the discovery of metal transporters and other biological participants by large-scale screenings [122,123].

#### 4.9.1. Copper and Lysyl Oxidases

Copper is essential for the functional activity of the lysyl oxidase (LOX) family of enzymes, which are notably responsible for the cross-linking and fibrillar stabilization of elastin and collagen in the extracellular compartment [124]. In humans, a deficiency in copper can lead to Menkes disease or occipital horn syndrome (OHS, also called X-linked cutis laxa or type 9 Ehlers-Danlos). These disorders have mild to severe forms and multiple organs can be affected [125]. Cardiovascular symptoms are not the major impact of these diseases, but abnormal vasculature and tortuosity with thin walled arteries were reported [125].

Human LOX family comprises five members, one LOX, and four lysyl oxidase-like (LOXL1-4) [126]. Eight LOX/LOXL isoforms have been identified in the zebrafish, with an ortholog for each human one, except for LOXL4 [127]. In human and in mammal models, deficiencies in LOX have been linked to an increased risk to develop aortic aneurysms and dissections [128]. However, in the zebrafish, they have only been studied in the context of notochord malformation [127,129] and early morphogenesis [130].

Menkes disease and OHS are often caused by a mutation in the *ATP7A* gene, which encodes a copper transporting ATPase, whose malfunction affects copper-dependent enzymes such as LOX/LOXL. A model of these diseases was developed in the zebrafish, based on the *calamity* mutant. The *calamity* mutant, resulting from the mutation of the ortholog of *ATP7A*, was characterized by an inadequate development of the notochord, as seen when LOXs were inhibited. It was described as phenotypically very close to chemically induced copper-deficient embryos, in which cartilage and vascular development were altered. Embryos showed disorganization of their intersegmental vessels, along with microaneurysm in some cases. However, these observations were not explicitly reported in the *calamity* mutant, so its vascular aspects remain to be ascertained [131].

In another study, a therapeutic strategy was tested to correct the *calamity* phenotype by targeting the wrongly-spliced mRNA by a morpholino-based approach and managed to rescue mutants in vivo [132].

Another mutant named *catastrophe* was found to affect copper metabolism in a chemical genetic screening study. It was associated to a mutation in a gene coding for a subunit of the vacuolar H+ ATPase (Atp6), which is not directly linked to copper metabolism [133]. This could lead to a better understanding of copper metabolism.

#### 4.9.2. Calcium and Vascular Mineralization

Lastly, another modification that can affect the vascular wall and elastic fiber behavior in particular is the mineralization of the media. Ions, mainly calcium, can accumulate abnormally in the vessel wall and damage elastic fibers. This is observed in aging vessels as an expected modification that occurs with time, but there are also pathologies displaying extensive calcification of the vascular wall that is not related to age. For example, in pseudoxanthoma elasticum (PXE), elastic fibers are fragmented because of their ectopic calcification, with dermatologic, ophthalmologic, and vascular consequences [134]. Concerning vascular dysfunction, PXE patients can develop arterial stenosis, tortuosity, and occlusion, as well as intracranial arterial aneurysm [134]. PXE has been linked to mutations in *ABCC6* and *ENPP1*. These genes are also involved in another disease called “generalized arterial calcification of infancy” or GACI, which is more severe with a life expectancy of only a few months. Zebrafish models have been developed to understand the consequences associated with the loss of both these genes, separately.

ABCC6 is a member of the ATP-binding cassette transporter family, whose role in mineralization is not entirely clear yet. The naturally occurring *gräte* zebrafish mutant, characterized by bones hypermineralization, was associated with the mutation of *abcc6a*, an ortholog of *ABCC6*. Morpholino and CRISPR/Cas9 approaches were also used to create other *abcc6a* mutants, but none of them recapitulate the complete phenotype of PXE, as ectopic mineralization was only rarely observed in zebrafish mutants [135]. Yet, *abcc6a* was found to be expressed in the eyes, heart, and intestines of young adult zebrafish [136]. More recently, another mutant was obtained by a TALEN-based approach with ocular calcification and fibrotic heart, the latter not being part of human PXE reported symptoms [136]. These differences of phenotype could be due to two reasons. First, *abcc6a* is not expressed in zebrafish liver, where it is thought to have an important role in mammals [137]. In addition, *abcc6a* has a paralog, *abcc6b*, that could reduce damages caused by *abcc6a* loss. A morpholino-based knockdown of *abcc6b* was tested alone, and revealed no phenotypic changes [138], but observations were restricted to embryos (until five days post-fertilization).

Some studies to find new therapeutic targets are also ongoing using *abcc6a* zebrafish mutants, for example with tests on vitamin K potential to reduce hypermineralization [139], or by correcting a mislocation of the Abcc6a transporter associated with certain PXE mutations [140].

Concerning the second gene associated with PXE, the zebrafish *enpp1* mutant, also called *dragonfish*, is characterized by an ectopic calcification of many tissues including soft ones. Enpp1 is an ectonucleoside pyrophosphatase/phosphodiesterase involved in the provision of pyrophosphate in the extracellular medium. In this mutant, ectopic mineralization was found in cartilage elements, skin, and in the cardiovascular system. Indeed, the vessels of embryos seemed to be normal but the observation of the bulbus arteriosus of adult zebrafish showed ectopic calcification. Osteoclast-like cells were also reported in the affected tissues [141].

### 4.10. Zebrafish Model Potential Contributions Depending on Developmental Stages

There are four main stages in zebrafish life-cycle: the embryo (until hatching; approximately 50 h post-fertilization), the larva (until metamorphosis; around 28 dpf), the juvenile (until the end of puberty; approximately 3 months post-fertilization), and the mature adult [142]. The cardiovascular system therefore undergoes different degrees of maturity corresponding to these developmental stages and a deficiency in elastic fibers will have distinct repercussions depending on these periods.

Zebrafish models presented in this review concern mostly experimentations made on embryonic and larval stages. These are interesting for the first steps of investigation, giving an overview of the effects of a mutation or a treatment on global development and general integrity of the organism, with mainly morphological and phenomenological observations. As mentioned previously in this review, the use of zebrafish embryos and larvae in human pathology research brings numerous advantages. First, they allow to monitor a whole organism response without requiring too many specific facilities (as in mouse experimentation, for example). Secondly, their transparency enables the observation of important developmental events in vivo, notably vasculogenesis and angiogenesis in the context of cardiovascular research, and blood vessel imaging has moreover been facilitated by the development of transgenic reporter lines such as Tg(*fli1a*:EGFP) [143] or Tg(*kdrl*:EGFP) [144], in which blood vessels appear fluorescent. Also, crossing those fluorescent strains with the transparent double mutant (*roy^−/−^*; *nacre*^−/−^) fish line casper enables to largely improve whole zebrafish imaging at early developmental stages [145]. Blood circulation can also be monitored in vivo by microangiography to highlight any defects [146] and basic hemodynamic parameters can be collected due to the developments of digital particle image velocimetry [147,148]. Lastly, the use of zebrafish larvae until 5 dpf is facilitated by ethic committees regarding animal experimentation issues, as they are not yet considered as “animals” per se and would therefore constitute a step in the direction of the 3R principles (Replacement, Reduction, Refinement) by reducing and replacing the mammal model use (European Commission Directive 2010/63/EU).

Despite these obvious assets, some limitations are also to be noted. As stated before, at early developmental stages, the maturity of the organism, including the cardiovascular system, has not been reached yet. Specific features such as the blood brain barrier, which takes place at the larval stage [149], or the coronary system, established after several weeks post-fertilization at the juvenile stage [150], would for example require the use of more mature models. In addition, the study of certain symptoms can be compromised, as well as severe outcomes over the long term that are impossible to consider.

The use of juvenile or adult zebrafish would be interesting in order to address these points, with the possibility of a more thorough analysis. As for larvae and embryos, numerous parameters can be measured to assess the cardiovascular function of juveniles and adults, such as weight and size of the heart and its different parts [151], but also heart rate and red blood cell flow rate [51]. Concerning vessels description and characterization, embryonic vessels are inappropriate to study histological organization and elastic mechanical properties because of their small size and immaturity. The use of older fish would be of greater interest for these concerns. In addition, the recent improvement of imaging techniques suitable for adult zebrafish is opening a new field of possibilities to visualize vessels in situ and potentially protein expression on a whole organism, with for example light sheet microscopy [152,153].

In the particular case of elastic fibers and elastin, the zebrafish model can offer a precious alternative, as the homozygous mutation of the elastin gene is rapidly lethal in mammal models (resulting in only a few days of life in mice [154]). The survival of mutant zebrafish is definitely interesting, but is also linked to the fact that elastin has been duplicated. It would be necessary to invalidate both paralogs to establish a strict comparison with the murine system. Indeed, one paralog could be able to compensate for the biological effects of the other as both paralogs are expressed jointly in almost all tissues [43]. However, the invalidation of only one paralog could provide a model to observe the impact of an elastic deficiency on the vascular architecture representing moderate forms of elastinopathies. The follow-up of heterozygous mutants is also possible, if necessary, and a comparison between homozygous mutants—for one or the other elastin gene—with a double heterozygous mutant could also be interesting, perhaps providing a greater variety of vascular outcomes due to the more or less severe phenotype.

## 5. Conclusions

Williams Beuren syndrome, non-syndromic supravalvular aortic stenosis, and the autosomal dominant cutis laxa are all consequences of mutations in the *ELN* gene. Patients suffering from these elastinopathies are predisposed to develop hypertension, arterial aneurysms, dissections, or stenosis. These cardiovascular conditions are also found in diseases affecting the other constituents of elastic fibers that form the microfibrils.

The development of new therapeutic strategies to treat these deadly diseases involves (i) a good understanding of the cardiovascular system at physiological and biochemical levels in humans and (ii) preliminary tests in animal models that recapitulate the disease and whose response to drugs will be as close as possible to that of humans.

In that way, the zebrafish present many advantages to study human pathologies in general and vascular diseases or malformations in particular. Indeed, zebrafish and human genomes share around 70% homology and most of the genes responsible for human cardiovascular diseases are conserved in zebrafish.

Despite some particularities, zebrafish and human cardiovascular systems are comparable and disease-model design based on pathological characteristics is possible due to the conservation of most of the mechanisms. Zebrafish is also perfectly suitable for genetic manipulations and the possibility to rapidly obtain a mutant model to test mutation consequences on a whole organism can be very practical as a first step of validation. Various models of fibrillinopathies reproducing elastic fiber defects found in human patients have been developed over the past decade and the recent release of *elastin* mutants through the Zebrafish Mutation Project will enable to mimic elastinopathies in the next few years.

The zebrafish is also well suited for the discovery of new drugs. High throughput chemical screening can easily be performed and collect more information on a real organism than the classical cell-based experiments. The possibility to test active compounds in vivo allows to study the interaction effects between the drug and the whole organism: potential toxicity, alteration by the metabolism, and pharmacokinetics and pharmacodynamics can for example be assessed.

Finally, the study of embryonic and larval zebrafish is easier compared to conventional rodent models as they are transparent and the visualization in 3D of adult circulatory system is now being resolved through the development of new imaging techniques such as light sheet microscopy.

All these points highlight the zebrafish model as a powerful tool to better understand elastic fiber-related vascular diseases, and more specifically, the zebrafish model represents a good ethical alternative for the first steps of investigations to reduce rodent usage.
